# Temperature-gradient incubation isolates multiple competitive species from a single environmental sample

**DOI:** 10.1099/acmi.0.000081

**Published:** 2019-12-02

**Authors:** Karen M. Houghton, Lucy C. Stewart

**Affiliations:** ^1^​ GNS Science, Wairakei Research Centre, 114 Karetoto Rd, Taupō 3384, New Zealand; ^2^​ GNS Science, 1 Fairway Drive, Avalon, Lower Hutt 5010, New Zealand

**Keywords:** Culture, Isolation, Environmental sample, Methanotroph, Temperature gradient, 16S rRNA

## Abstract

High-throughput sequencing has allowed culture-independent investigation into a wide variety of microbiomes, but sequencing studies still require axenic culture experiments to determine ecological roles, confirm functional predictions and identify useful compounds and pathways. We have developed a new method for culturing and isolating multiple microbial species with overlapping ecological niches from a single environmental sample, using temperature-gradient incubation. This method was more effective than standard serial dilution-to-extinction at isolating methanotrophic bacteria. It also highlighted discrepancies between culture-dependent and -independent techniques; 16S rRNA gene amplicon sequencing of the same sample did not accurately reflect cultivatable strains using this method. We propose that temperature-gradient incubation could be used to separate out and study previously ‘unculturable’ strains, which co-exist in both natural and artificial environments.

## Introduction

Over the last few decades, high throughput or next-generation sequencing (NGS) technologies have generated billions of 16S rRNA gene amplicon sequences with rapidly decreasing costs, allowing the detection of an incredible diversity of micro-organisms in almost every environment [1]. Sequencing has revealed a huge diversity of candidate taxa, which have yet to be cultivated, compared to the relatively limited set of microbes held in axenic culture [2]. However, sequencing data, absent confirmatory cultivation, is not an unbiased approach. There are known biases during the DNA extraction, amplification and sequencing processes [3–5], including the fact that amplification primers based on known consensus sequences may not bind to more novel gene sequences. Differences in sequencing and analysis protocols between laboratories can also make it difficult to directly compare sequence-based diversity studies.

Once obtained, the relative abundance of 16S rRNA or functional gene sequences can be associated with physiological state such as the disease status of a host, or correlated with environmental factors. However, they are not a measure of the activity of microbial cells, as it is difficult to determine if the DNA sequenced was obtained from live cells or extracellular or relic DNA [6]. Relic DNA can confound estimates of microbial abundance and diversity [7], although viability indicators, which limit amplification of extracellular DNA, such as propidium monoazide [8], can be used to differentiate between live and dead cells. Amplicon sequencing using current NGS platforms such as Illumina HiSeq or Ion Torrent produces relatively short reads (150–400 bp), which means it is difficult to assign taxonomy accurately to species level [9]. Different classifiers such as RDP [10], silva [11] or Greengenes [12] classify certain sequences differently, based in part on the historic use of phenotype-based classification [13]. In addition, it is difficult to detect microbes of low abundance (<10^6^ c.f.u. ml^−1^) [14] in high-biomass environments such as the human gut or soil, unless a sufficiently high coverage of sequencing is used [15], which is difficult to predict in advance [16]. Therefore, when a specific functional group or species is being investigated, sequencing can be less sensitive than targeted culturing methods [14]. Whole-metagenome and -metatranscriptome shotgun sequencing can allow taxonomy classification down to strain level with the use of multiple concatenated housekeeping genes [17], and can also identify functional genes, providing a direct link to potential microbial activity without relying on phylogeny as a predictor [18]. However, functional gene predictions must still be verified by cultivation and characterization of (ideally) axenic strains, particularly for novel groups, which do not necessarily use known genes or pathways for functions of interest [19, 20].

Sequencing became popular as an alternative to cultivation because cultivation is time-consuming. Difficulties in replicating environmental conditions have led to the ‘Great Plate Count Anomaly’ [21], where the number of observed microbial cells in an environmental sample surpasses the number that can be grown in the laboratory. To circumvent this issue, microbial culturomics was devised [14] – the application of multiple culture conditions to individual environmental samples to rapidly cultivate and identify large numbers of isolates.

Culturomics has been applied to isolate multiple novel micro-organisms from microbiomes including the guts of humans [22], gorillas [23], cows [24] and chickens [25], as well as environmental samples from a biogas plant [26] and solar saltern [27]. These studies generally involve plating samples on a wide range of media, while other studies have used nutrient gradients [28, 29] or pH gradients to separate microbes [30]. To our knowledge, there have been no attempts at using thermal gradients to isolate numerous different micro-organisms. In this study we chose to focus on aerobic methane-oxidizing bacteria (methanotrophs), which are found in a wide variety of environments, including geothermal systems. They are important not only for their ability to oxidize a significant greenhouse gas, but also for a wide variety of biotechnology applications, including bioremediation [31] and biopolymer and protein production [32, 33]. Pure cultures of methanotrophs are difficult to obtain, as many species are intolerant to agar and have slow growth rates. Cultures are easily contaminated with methylotrophic (methanol-oxidizing) or other heterotrophic bacteria [34]. Novel culturing techniques are required to isolate new methanotrophic strains, enabling optimization of growth conditions for development of novel products and processes.

In this proof-of-concept experiment, we incubated environmental hot spring samples at multiple temperatures, in order to isolate aerobic methanotrophs with overlapping ecological niches, which compete for the same resources in their natural habitat. We compared this method to conventional serial dilution to determine the effectiveness of the approach, and also to 16S rRNA gene sequencing of the same sample to validate the accuracy of culture-independent sequencing in identifying the breadth of methanotroph diversity in this environment. We suggest that this method could be adapted to allow cultivation and characterization of other novel strains with overlapping ecological niches.

## Methods

### Sampling

**Fig. 1.**
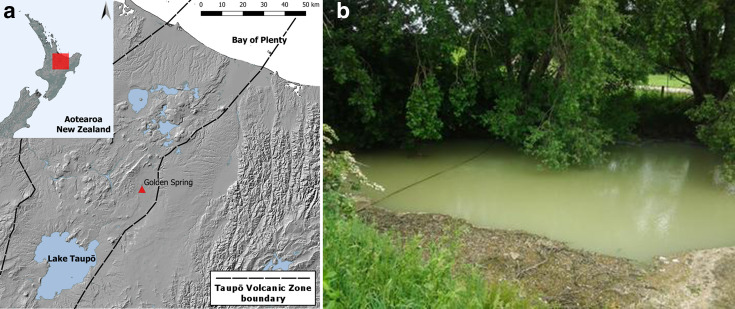
(a) Sampling location within New Zealand. The sampling site is indicated with a red triangle; (b) photograph of the sampling site, taken from [34].

The sampling site (Fig. 1) was identified from the 1000 Springs dataset [35] as having high *in situ* levels of methane (31 µM) and a high proportion of 16S rRNA gene sequences assigned to known methanotroph-containing genera (32.8 % identified as *Methylococcales *). An autoclaved 250 ml centrifuge tube was used to collect a sample from the water column, and a 50 ml tube was used to collect a sample of sediment at the bottom of the hot spring directly underneath the location of the water sample. Environmental samples were kept refrigerated for 2–3 days prior to extraction of DNA and inoculation into culture medium.

### 16S rRNA sequencing of environmental samples

DNA was extracted from the soil and water samples using a modified protocol for the NucleoSpin Soil kit (Macherey-Nagel, Germany) (Supplementary Materials, available in the online version of this article). The V4 region of the 16S rRNA gene (~300 bp) was amplified using universal primers and adaptor sequences for the Illumina MiSeq: 515F [36] and 806R [37]. Each sample was amplified in triplicate, pooled and purified using the NucleoSpin Gel and PCR Clean-up kit (Macherey-Nagel) and Agencourt AmPure XP (Beckman Coulter, IN, USA). Amplicon libraries were prepared and sequenced by Macrogen (South Korea). The data have been deposited with links to BioProject accession number PRJNA546003 in the NCBI BioProject database (https://www.ncbi.nlm.nih.gov/bioproject/).

The quality of raw read data was assessed using FastQC [38]. Paired-end sequence reads were merged and filtered using USEARCH v7.0, with a maximum expected error of 1 [39]. Remaining sequences either >500 bp (to remove poor-quality sequences at the end of long reads) or <200 bp (the minimum required for taxonomy classification) were removed using mothur v1.35.1 [40]. A *de novo* database of ≥97 % similar sequence centroids or operational taxonomic units (OTUs) was created in USEARCH [39]. Raw sequences were mapped against this *de novo* database to generate counts of sequences matching OTUs (i.e. taxons) for each sample. Using QIIME v1.9.1 [41], taxonomy was assigned to each OTU by using the RDP Classifier v2.2 [42] trained on the Silva 16S rRNA gene database (version 123) [43] with a confidence threshold of 0.5. Chloroplast and mitochondrial OTUs were removed and both samples were rarefied to 299 600 reads per sample.

All OTUs identified by the RDP Classifier as belonging to the methanotrophic families *Beijerinckiaceae* or *Methylocystaceae* (* Alphaproteobacteria *), the order *Methylococcales* (* Gammaproteobacteria *), or the genus *Methylacidiphilum* (* Verrucomicrobia *) were manually checked against the NCBI database using a discontiguous megablast [44]. OTUs that were >90 % related to a described methanotrophic species were identified as putative methanotrophs. Although there are no universal definitions for higher taxa using 16S rRNA gene sequences, the 90 % similarity level is commonly used to denote the boundary of an order within a phylum [45, 46]. Alpha rarefaction was performed in QIIME using chao1, observed OTUs and Shannon diversity metrics.

### Thermal gradient and dilution-to-extinction cultivation

For all cultivation experiments, 0.25 g of both of the environmental samples were inoculated into nitrate mineral salts media (NMS) [47] with an air headspace with the addition of approximately (v/v) 8 % CH_4_ and 0.08 % CO_2_. For dilution-to-extinction, duplicate samples were incubated at 37 °C with shaking at 150 r.p.m. For thermal-gradient experiments, 12 duplicate tubes were incubated in a temperature-gradient incubator (Terratec Corporation, Hobart, Australia), set to 17–57 °C (the *in situ* sample temperature of 37 °C±20 °C) and agitated at 70 oscillations per minute. Methane oxidation from turbid cultures was observed via headspace sampling and quantification by gas chromatography using a flame ionization detector (GC-FID) (Peak Performer 1; Peak Laboratories, CA, USA). Enrichment cultures were transferred to fresh media (1 : 10 v/v) and incubated under the same conditions. After at least three passages with concurrent methane oxidation, the enrichments were serially diluted to extinction. The antibiotic monensin (30 µg ml^−1^), which specifically targets Gram-positive micro-organisms, was used to remove contaminating bacterial species in mixed cultures. The resultant cultures were checked for axenic purity via phase-contrast microscopy (Eclipse Ni; Nikon, Tokyo), the absence of growth of heterotrophs on complex media, and sequencing of the 16S rRNA gene.

### Genomic DNA extraction

Genomic DNA was extracted from the isolated methanotrophs using a NucleoSpin Tissue kit (Macherey Nagel) according to the manufacturer’s instructions. The 16S rRNA gene was amplified using the universal bacterial primers 9F and 1492R [48]. Particulate methane monooxygenase genes were targeted using the forward primer A189b [49] with one of the reverse primers A650 [50], mb661 [49] or A682 [51]. The primer pair mmoXA and mmoXB [52] were used to amplify the soluble methane monooxygenase *mmoX* gene. PCR reactions that produced a product of the expected size (~1400 bp for the 16S rRNA gene, ~500 bp for *pmoA* and ~1200 bp for *mmoX*) were cleaned using the NucleoSpin Gel and PCR Clean-up kit (Macherey-Nagel), before subsequent sequencing by Macrogen (South Korea).

### Phylogenetic analysis

The near-full length 16S rRNA gene sequences were manually checked for quality. Closely related strains were determined by conducting a pairwise similarity assessment of the isolate 16S rRNA gene sequences using blast (discontiguous megablast search) [44] in the NCBI portal. The 16S rRNA gene sequences from the isolates and closely related strains were aligned (all retrieved sequences were >1355 bp in length) using the clustal W algorithm in mega7 [53]. Phylogenetic distances were inferred by using the maximum-likelihood methodology [54] based on the Jukes–Cantor model [55]. The consensus tree was inferred from 1000 bootstrap replications. Initial tree(s) for the heuristic search were obtained automatically by applying neighbour-joining and BioNJ algorithms to a matrix of pairwise distances estimated using the maximum composite likelihood [56] approach, and then selecting the topology with superior log likelihood value. The GenBank/EMBL/DDBJ accession numbers for the 16S rRNA gene sequences of the isolates are MN511716–MN511723.

## Results

### 16S rRNA sequencing of environmental samples

**Fig. 2.**
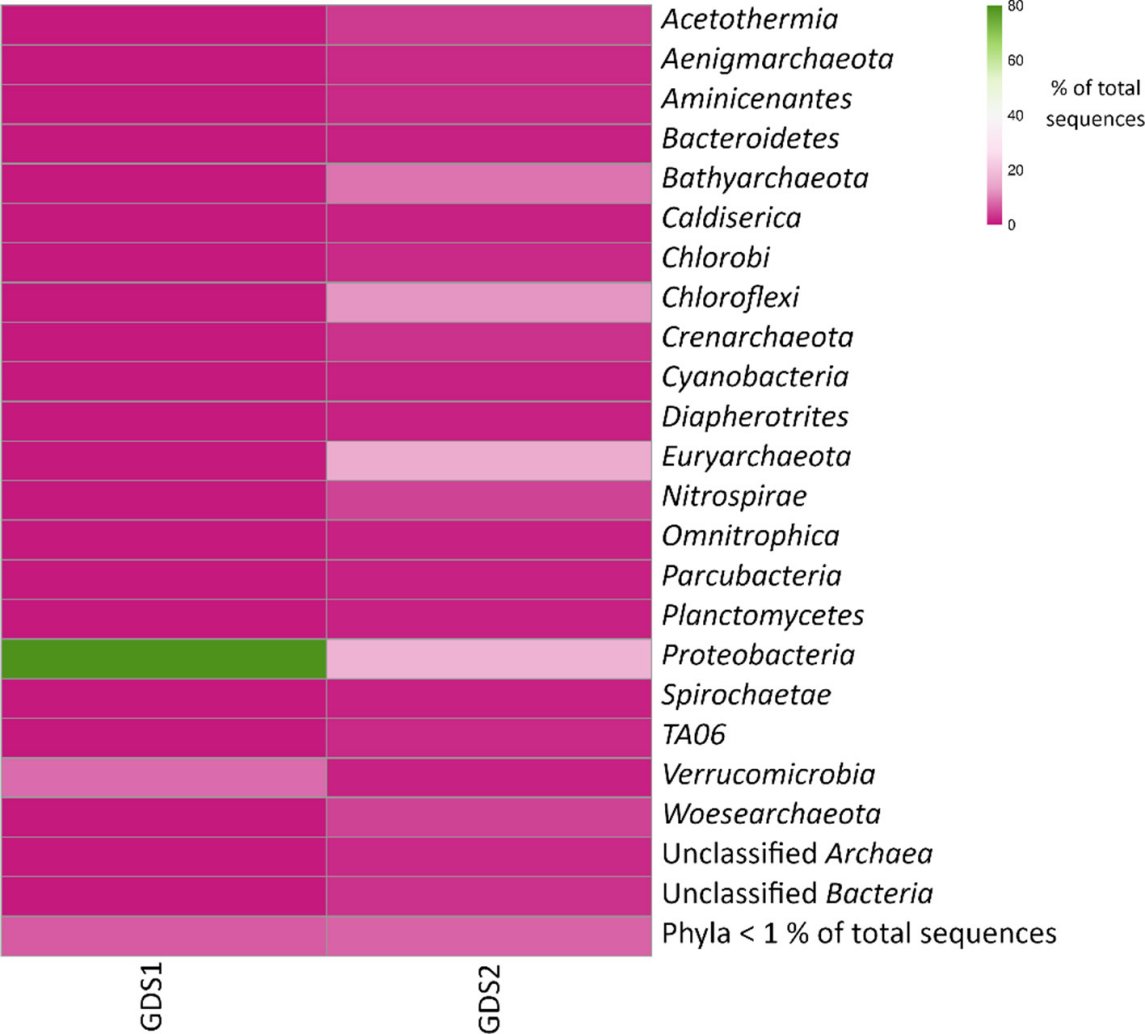
Distribution of 16S rRNA sequences by phyla from the water (GDS1) and sediment (GDS2) samples.

Sequences were rarefied to 299 600 reads per sample. Rarefaction curves (not shown) indicated that the samples had reached near saturation at this sequencing depth, according to the observed number of OTUs as well as estimates of richness (Chao1) and diversity (Shannon). The GDS1 (water) sample contained 1422 OTUs and was dominated (79.2 %) by reads classified as *Proteobacteria* (Fig. 2), with 50.4 % of these within *Pseudomonadales *. As previously predicted from other studies [57], the sediment sample was more diverse than the water column, with 3022 unique OTUs identified. The most abundant phylum was likewise *Proteobacteria* (17.4 %), although *Euryarchaeota* (15.5 %) and *Chloroflexi* (12.1 %) OTUs were also abundant in this sample (Fig. 2).

**Fig. 3.**
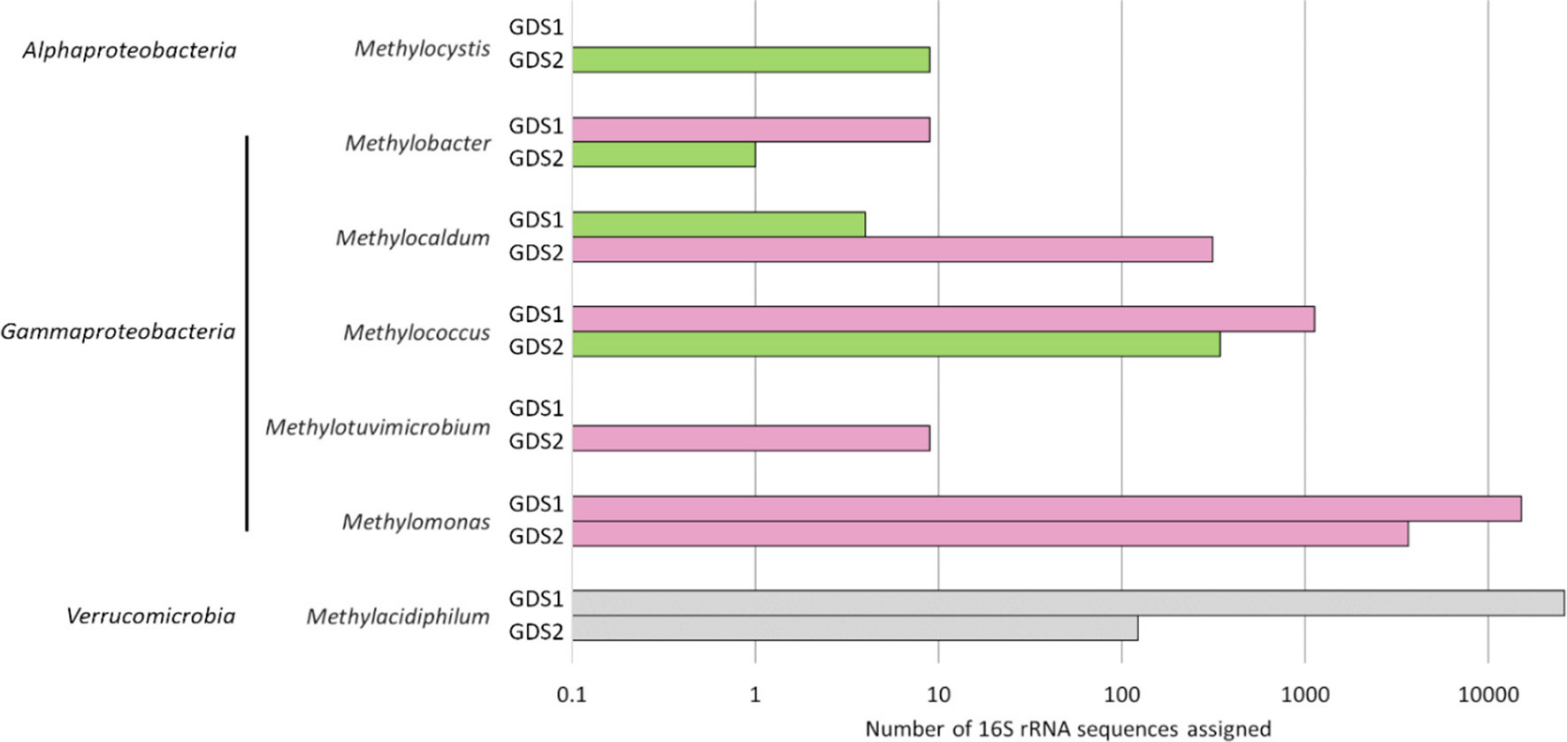
Graph of the number of 16S rRNA amplicon sequences assigned to each methanotrophic genus from the two sample sites. The bar colour indicates whether methanotroph strains from that genus were also cultivated from that site: green, successful cultivation: pink, unsuccessful cultivation; grey, cultivation not attempted. The number of strains cultivated is given in Table 1.

Putative methanotrophs were identified within both samples, with the GDS1 sample having 5.4 % of all reads assigned to the order *Methylococcales* and 8.7 % to *Methylacidiphilales*. These were classified in the genera *Methylobacter *, *Methylocaldum *, *Methylococcus* and *Methylomonas (Methylococcales*) or *Methylacidiphilum (Methylacidiphilales*) (Fig. 3). The GDS2 sediment sample had much lower abundances of methanotroph sequences, and included nine reads each classified as *Methylocystis* or *Methylotuvimicrobium *, which were not identified in the water sample.

**Table 1.** Aerobic methanotroph strains isolated in this study

Isolate	Morphology	Isolation temperatures (°C)	Growth temperature range (°C)	Genus
GDS1.2	Cocci-bacilli	35, 37	22–46	* Methylocystis *
GDS1.3	Short rods	17, 25, 27	17–37	* Methylosinus *
GDS1.7	Vibrio	37	20–45	* Methylocystis *
GDS1.8	Cocci	35, 38, 41	24–46	* Methylocaldum *
GDS1.10	Short, thick rods	41	24–45	* Methylocystis *
GDS2.4	Cocci	37, 41, 45	27–49	* Methylococcus *
GDS2.7	Vibrio	28, 37, 43	17–44	* Methylocystis *
GDS2.14	Short rods	24, 28, 31	18–41	* Methylobacter *

### Standard dilution-to-extinction cultivation

Samples of both water and sediment from the Golden Springs site were inoculated into NMS medium, which has been previously used to culture multiple methanotrophs including *Methylomonas *, *Methylococcus* [47, 58] and *Methylobacter* [59] strains, and incubated at 37 °C. After less than a week of incubation, both enrichment cultures became visibly turbid and methane oxidation was detected. They were serially diluted multiple times until an isolate was obtained from each.

Strain GDS1.7 was isolated from the water sample, and appeared as non-motile curved rods, approximately 0.8 µm wide by 2–4 µm long. Cells grew between 20–43 °C, with an optimum of 36 °C. The 16S rRNA gene sequence of GDS1.7 was most closely related (100 % sequence identity) to *Methylocystis* KS8a, a strain isolated from freshwater lake sediment in Israel but not fully characterized [60]. Strain GDS2.4 was isolated from the sediment sample. Cells were non-motile and coccoid, approximately 0.8–2.0 µm in diameter, and were usually seen in pairs. Cultures grew between 27–49 °C with an optimum of 40 °C. The 16S rRNA gene sequence of GDS2.4 shared 97 % nucleotide identity with *Methylococcus capsulatus* str. Bath, which was detected in the Illumina sequencing, although this was of low abundance (344 reads / 0.1 %).

### Thermal-gradient enrichment and cultivation

To determine if thermal-gradient incubation could be used to improve isolation efficiency, identical samples of Golden Springs water and sediment were incubated in NMS media over a gradient of 40 °C (17–57 °C). Over a period of 2 weeks, methane oxidation and a range of cell morphologies were observed in tubes incubated at 17–47 °C. No growth or methane oxidation was seen in tubes incubated between 47 and 57 °C. Each turbid culture was inoculated into fresh media multiple times and maintained at the same incubation temperature, with consequent turbid cultures then serially diluted to extinction. This culturing strategy resulted in some strains being isolated multiple times from enrichment cultures incubated at different temperatures (Table 1).

**Fig. 4.**
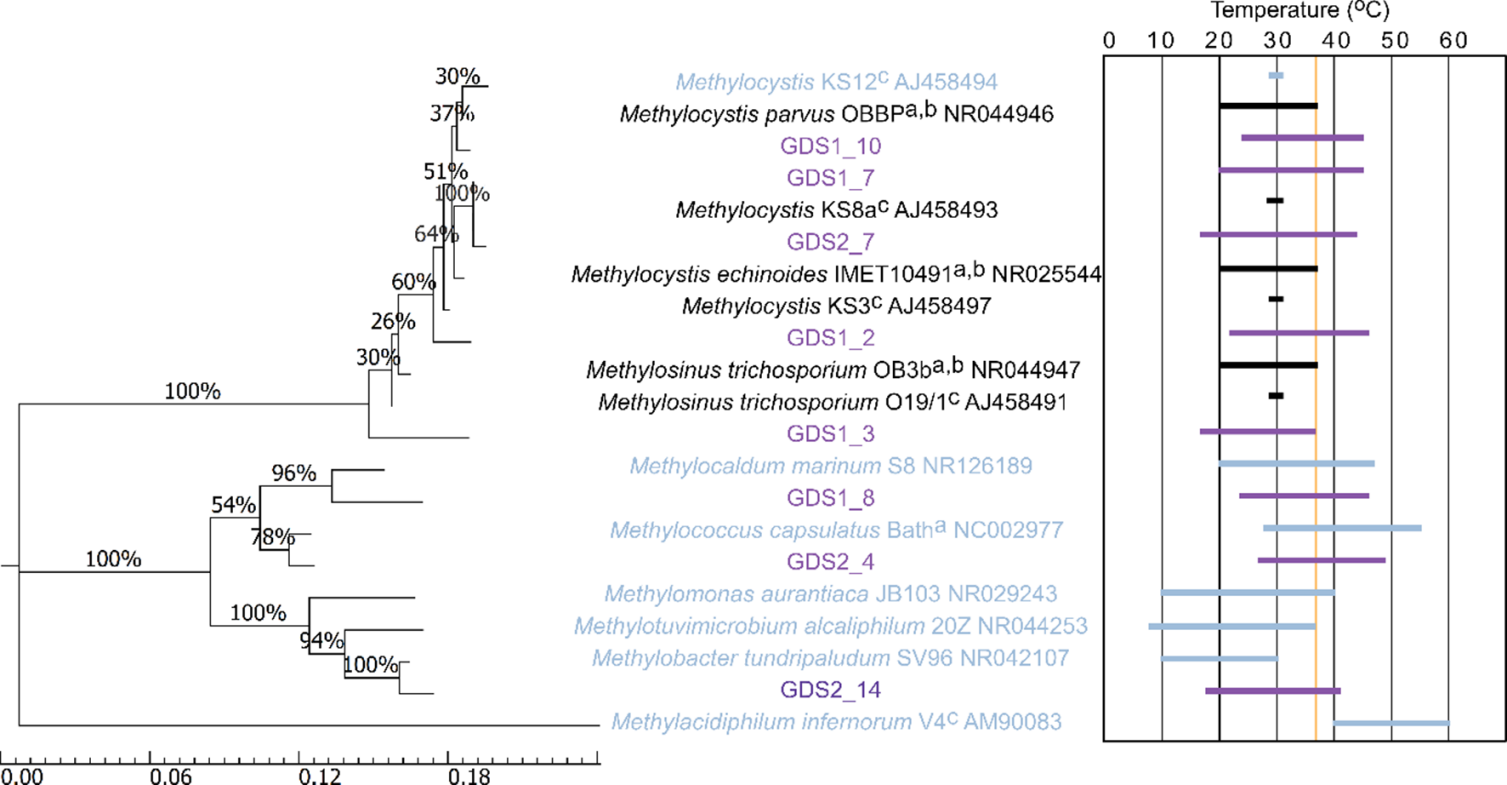
Molecular phylogenetic analysis of 16S rRNA gene sequences from isolated methanotrophs and closely related strains, with growth temperature ranges for each. The temperature of the original sample (37 °C) is highlighted with a yellow line. Purple names and bars indicate strains isolated in this study; blue names and bars indicate strains identified through Illumina sequencing of DNA extracted from the Golden Springs site; black names and bars indicate closely related strains not identified through sequencing. a, no *T*_min_ published; b, no *T*_max_ published; c, only growth temperature published. The evolutionary history was inferred using the aximum-likelihood method based on the Jukes–Cantor model [55]. The percentage of replicate trees in which the associated taxa clustered together in the bootstrap test (1000 replicates) is shown next to the branches. The scale bar represents nucleotide substitutions per site. Evolutionary analyses were conducted in mega7 [53].

Near full-length 16S rRNA gene sequences from all isolates indicated that the GDS1.7 *Methylocystis* and GDS2.4 *Methylococcus* strains were re-isolated using the gradient-temperature incubation, although the GDS1.7 strain was only identified in one culture. Other strains isolated using this approach included *Methylosinus *, *Methylocaldum* and *Methylobacter* strains, which were all capable of growth at 37 °C but had varying temperature ranges for growth (Fig. 4). The *Methylosinus* (GDS1.3) and *Methylobacter* (GDS2.14) strains were both relatively slow growing, taking up to 2 weeks for cultures to become turbid. Sequencing of particulate methane monooxygenase (*pmoA*) genes from all strains indicated very similar phylogeny to the 16S rRNA gene sequences (Fig. S1). Three strains also contained the rarer soluble methane monooxygenase (*mmoX*) gene (Fig. S2).

## Discussion

We chose our sample site because the previous 1000 Springs report [35] suggested a high abundance of aerobic methanotrophs. Compared to that study, we detected fewer methanotroph-associated sequences in this pond, perhaps demonstrating temporal variability. The profusion of methanotrophs in the water sample was surprising given the low solubility of methane in water [61], while the lower abundances recorded in the sediment may reflect slow diffusion rates of methane through sediment pores [62].

Thermal-gradient incubation was more effective than the standard dilution-to-extinction method at isolating novel strains, with nine strains isolated from a range of temperatures compared to only two isolates when samples were incubated at the *in situ* temperature of 37 °C. The temperature-gradient incubation also highlighted the disparity between the culture-independent method and cultivation, with several strains not identified in the NGS sequencing of 16S rRNA genes from the hot spring able to be isolated from the same sample. The water sample (GDS1) isolates included a *Methylosinus* strain and three *Methylocystis* strains, neither genus of which was detected by 16S rRNA amplicon sequencing, in addition to a *Methylocaldum* strain, which was represented by just four reads (0.001 %). The GDS2 sediment sample had only nine reads (0.003 %) classified as *Methylocystis* and one as *Methylobacter *, but three strains from these two genera were isolated. In contrast, *Methylomonas *, which was represented by 15 129 and 3644 reads in the water and sediment samples, respectively (5.0 and 1.2 %), was not cultured during this study, despite the same media being previously used to cultivate other *Methylomonas* species [47, 58]. This may be due to the short ‘shelf-life’ of many *Methylomonas* strains, which are not desiccation- or heat-resistant and respond poorly to periods of methane starvation [63]. Two slow-growing cultivated strains – a *Methylosinus* and a *Methylobacter* – were able to be isolated at lower temperatures than their original environment (17–27 and 24–31 °C, respectively) where they could not be out-competed by faster-growing methanotrophs with higher-temperature optima, such as *Methylococcus* [47]. *Methylococcus* was isolated from the sediment sample but not the water sample, even though sequences associated to this genus were detected in both samples; the cultivation method may not have been optimal for this genus in ways we are not aware of.

Reads assigned to *Methylacidiphilum infernorum* comprised 8.7 % of reads from the GDS1 water sample and were identified at low abundance in the GDS2 sediment sample. We did not target *Methylacidiphilum* species for enrichment as the temperature and pH of both samples (37 °C, pH 7.4) were outside the known growth ranges for this strain (40–60 °C, pH 1.0–6.0) [64]. While it is possible that extracellular or relic DNA [6] was sampled during this study, a known issue when measuring community richness and diversity [7], we cannot assess whether live cells were present or not, as we did not perform culture experiments within its known pH range. It may be dormant, growing in low-pH microclimates within the pond, or these sequences may represent a related species with less stringent growth constraints, which was not competitive with the strains we isolated under conditions of neutral pH. A multiple-variable set of gradient experiments using temperature *and* pH could be used to investigate this question.

The isolation of three strains (GDS1.3 *Methylosinus *, GDS1.8 *Methylocaldum* and GDS2.4 *Methylococcus *), which contain a soluble methane monoxygenase (sMMO), in addition to the more common particulate or membrane-bound form of the enzyme (pMMO), may be related to the concentration of copper in the samples. While expression and activity of pMMO intensifies as copper concentrations increase [65], the sMMO is only expressed under low copper conditions, giving a selective advantage to methanotrophs able to switch between the two forms of the enzyme. The concentration of copper in the Golden Springs water in the previous 1000 Springs study was 0.2 p.p.m. [35], meaning that sMMO may be the dominant form of methane monooxygenase used in this ecosystem, and strains which do not possess sMMO may not be competitive.

### Conclusions

This proof-of-concept study demonstrates that thermal-gradient cultivation is a more effective method of isolating bacteria from environmental samples than standard dilution-to-extinction protocols. It separates microbes based on their temperature optima and reduces competition, which allows isolation of slow-growing strains, which would be out-competed by fast growers in the usual culturing process. We propose that this cultivation method could be adapted for the enrichment and isolation of novel microbial strains from other environments, which experience temperature changes and gradients, such as other geothermal or hydrothermal environments, those in locations with freeze–thaw cycles, and desert climates. Thermal-gradient incubation can also be easily and usefully combined with gradients of other conditions without affecting chemistry. A gradient of copper concentration could be used to separate methanotrophs, which contain the soluble form of methane monooxygenase (repressed by high copper) from those expressing the particulate form. Both forms of the enzyme oxidize a wide of substrates, including organic pollutants, in addition to methane, and isolates can then be screened for their bioremediation potential. For other metabolic clades, metagenomic data could be used to suggest nutrient gradients, or increasing concentrations of antibiotics where targeted microbes have antibiotic resistance markers.

This study confirms the importance of validating hypotheses from sequencing data with cultivation experiments, and that low abundance members of the community, which may be missed entirely if the sequencing is not of sufficient depth, can still play important ecological roles [66–68]. Targeted culture experiments greatly expand the ability to identify novel compounds such as new antibiotics, and new pathways for biotechnology and bioremediation.

## Supplementary Data

Supplementary material 1
