# Supplementary material for: Temperature-gradient incubation isolates multiple competitive species from a single environmental sample
Source: Access Microbiol. 2019 Dec 2;2(3):acmi000081. doi: 10.1099/acmi.0.000081 (PMC7470311; doi:10.1099/acmi.0.000081)
Supplement: Supplementary material 1 [file acmi-2-081-s001.pdf]

Supplementary Information

DNA was extracted from soil and sediment microcosms using a modified protocol for the NucleoSpin Soil kit (Macherey-Nagel, Germany). 0.25 g of sample was placed into bead beating tubes along with 200 µl of Difco™ sterile skimmed milk solution (5 % w/v), 150 µl Enhancer SX and 700 µl of either Lysis Buffer SL1 or SL2, provided with the kit. Cells had two cycles of disruption in a bead beater (Biospec, OK) at maximum speed for 30 seconds followed by cooling at 4 °C for > 5 minutes. Samples were next centrifuged at 11,000 x *g* for one minute and the supernatant removed to a 2 ml microfuge tube. Lysis Buffer SL3 (150 µl) was then added, the tubes were briefly vortexed and centrifuged again at 11,000 x *g* for one minute and the supernatant transferred into a new tube. 125 µl of a 7.5 M potassium acetate solution was added and vortexed and the resultant mix was centrifuged at 17,000 x *g* for five minutes. Finally, the supernatant was removed to a new tube containing 250 µl of Binding Buffer SB and vortexed briefly before being applied to a spin filter column and centrifuged at 11,000 x *g* for one minute. The remainder of the manufacturer's protocol was then followed, with the exception that DNA was eluted twice with 40 µl Elution Buffer SE heated to 70 °C.

Cells were grown in NMS medium in 18 mm (dia.) anaerobic tubes sealed with gas-tight butyl rubber stoppers. The tubes were incubated in duplicate in a Temperature Gradient Incubator (Terratec Corporation, Hobart, Australia), initially set to a Δ 40 °C gradient (± 20 °C initial sample temperature) and agitated at 70 oscillations per minute. OD_{600} was measured daily using an adaptor (Perkin-Elmer, Waltham, MA) that allowed the tubes to be directly inserted into a Halo Vis-10 spectrophotometer (Dynamica Scientific, Newport Pagnell, UK) without subsampling cultures. The optimum temperature for growth was estimated based on the fastest growth rate and the highest OD obtained for each strain.

The *pmoA* sequences from the isolates (all sequences were > 448 bp in length) and *mmoX* sequences (> 1140 bp) were aligned with sequences obtained from the GenBank database, in MEGA7 (1). Derived amino acids were determined via MEGA7 and used to construct phylogenetic trees using the Maximum Likelihood method based on the JTT matrix-based model (2). The consensus tree was inferred from 1000 bootstrap replications. Initial trees(s) for the heuristic search were obtained automatically by applying Neighbour-Joining and BioNJ algorithms to a matrix of pairwise distances estimated using a JTT model, and then selecting the topology with superior log likelihood value.

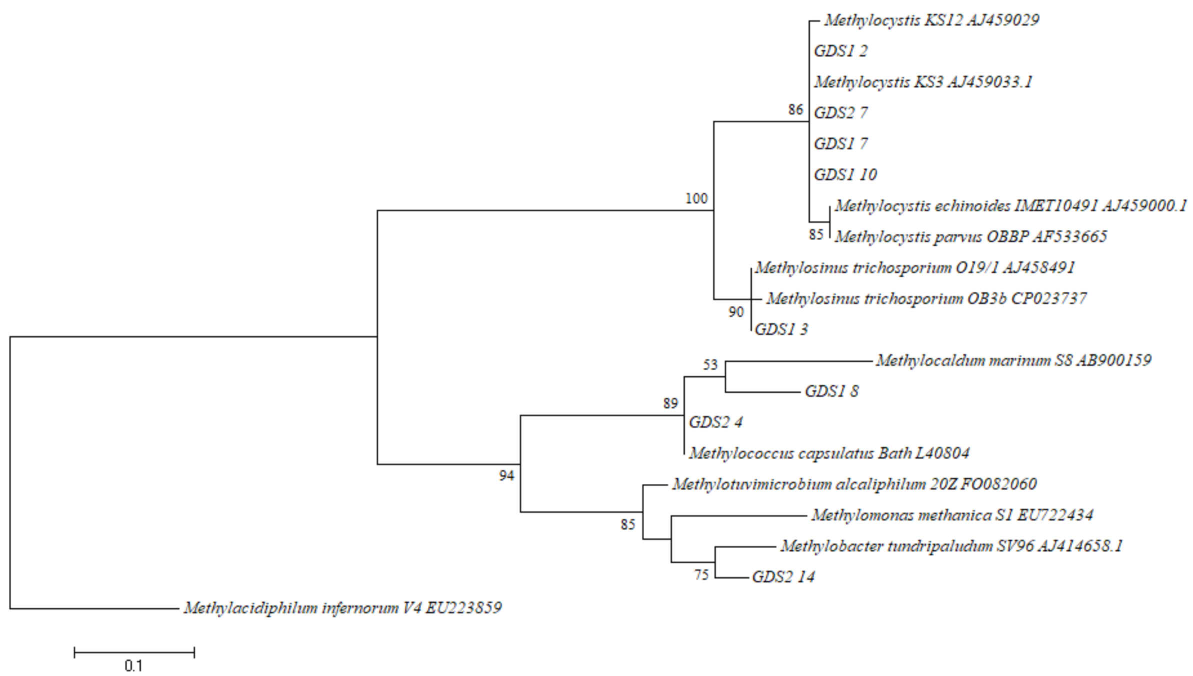

Supplementary Figure 1. Maximum likelihood PmoA sequence phylogeny of isolates. The tree shows the phylogenetic relationships between GDS isolates and closely related methanotrophic isolates, based on the inferred amino acid sequence of PmoA. Bootstrap values > 50 % are shown at the branch nodes (1000 replicates). The scale bar indicates the number of amino acid substitutions per site.

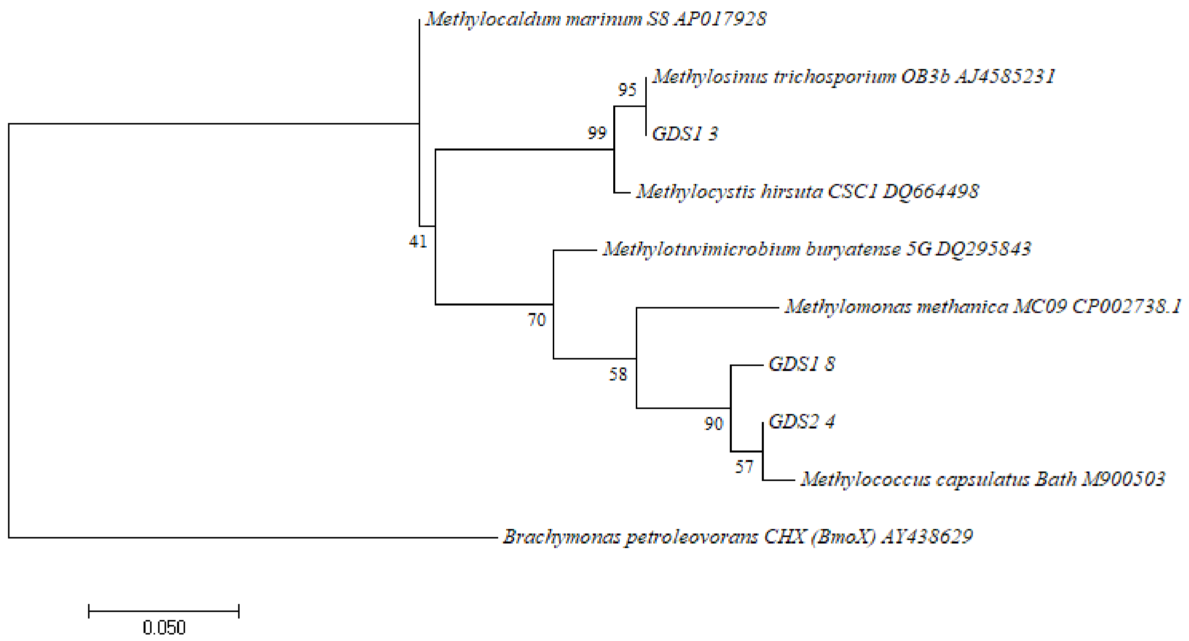

Supplementary Figure 2. Maximum likelihood MmoX sequence phylogeny of isolates. The tree shows the phylogenetic relationships between GDS isolates and closely related methanotrophic isolates, based on the inferred amino acid sequence of MmoX. Bootstrap values > 50 % are shown at the branch nodes (1000 replicates). The scale bar indicates the number of amino acid substitutions per site.

1. Kumar S, Stecher G, Tamura K. MEGA7: Molecular Evolutionary Genetics Analysis version 7.0 for bigger datasets. *Mol Biol Evol*. 2016;33(7):1870-4. 10.1093/molbev/msw054
2. Jones DT, Taylor WR, Thornton JM. The rapid generation of mutation data matrices from protein sequences. *Bioinformatics*. 1992;8(3):275-82. 10.1093/bioinformatics/8.3.275
